# Development of a new risk stratification system for patients with newly diagnosed multiple myeloma using R-ISS and ^18^F-FDG PET/CT

**DOI:** 10.1038/s41408-021-00577-2

**Published:** 2021-12-01

**Authors:** Hee Jeong Cho, Sung-Hoon Jung, Jae-Cheol Jo, Yoo Jin Lee, Sang Eun Yoon, Sung-Soo Park, Do Young Kim, Ho-Jin Shin, Yeung-Chul Mun, Jun Ho Yi, Hyo Jung Kim, Da Jung Kim, Ho Sup Lee, Sung Hwa Bae, Chae Moon Hong, Shin Young Jeong, Jung-Joon Min, Sang Kyun Sohn, Chang-Ki Min, Kihyun Kim, Je-Jung Lee, Joon Ho Moon, The Korean Multiple Myeloma Working Party, Hee Jeong Cho, Sung-Hoon Jung, Jae-Cheol Jo, Yoo Jin Lee, Sang Eun Yoon, Sung-Soo Park, Do Young Kim, Ho-Jin Shin, Yeung-Chul Mun, Jun Ho Yi, Hyo Jung Kim, Da Jung Kim, Ho Sup Lee, Sung Hwa Bae, Chae Moon Hong, Shin Young Jeong, Jung-Joon Min, Sang Kyun Sohn, Chang-Ki Min, Kihyun Kim, Je-Jung Lee, Joon Ho Moon

**Affiliations:** 1 Department of Hematology-Oncology, Kyungpook National University Hospital, School of Medicine, Kyungpook National University, Daegu, South Korea; 2 Department of Hematology-Oncology, Chonnam National University Hwasun Hospital and Chonnam National University Medical School, Hwasun-gun, Jeollanam-do South Korea; 3 Department of Hematology and Oncology, Ulsan University Hospital, University of Ulsan College of Medicine, Ulsan, Korea; 4 Department of Medicine, Sungkyunkwan University School of Medicine, Samsung Medical Center, Seoul, South Korea; 5 Department of Hematology, Seoul St. Mary’s Hospital, College of Medicine, The Catholic University of Korea, Seoul, South Korea; 6 Department of Hematology-Oncology, Pusan National University Hospital, Pusan National University School of Medicine, Busan, South Korea; 7 Department of Hematology-Oncology, Ewha Womans University Medical Center, Seoul, South Korea; 8 Department of Hematology-Oncology, Chung-Ang University Hospital, Seoul, South Korea; 9 Department of Hematology-Oncology, Hallym University Sacred Heart Hospital, Anyang, South Korea; 10 Department of Hematology-Oncology, Kosin University Gosper Hospital, Busan, South Korea; 11 Department of Hematology-Oncology, Daegu Catholic University Hospital, Daegu Catholic University School of Medicine, Daegu, South Korea; 12 Department of Nuclear Medicine, Kyungpook National University Hospital, School of Medicine, Kyungpook National University, Daegu, South Korea; 13 Department of Nuclear Medicine, Chonnam National University Hwasun Hospital and Chonnam National University Medical School, Hwasun-gun, Jeollanam-do South Korea

**Keywords:** Myeloma, Risk factors

## Abstract

In multiple myeloma (MM), a high number of focal lesions (FL) detected using positron emission tomography/computed tomography (PET/CT) was found to be associated with adverse prognosis. To design a new risk stratification system that combines the Revised International Staging System (R-ISS) with FL, we analyzed the data of 380 patients with newly diagnosed MM (NDMM) who underwent ^18^F-fluorodeoxyglucose (^18^F-FDG) PET/CT upon diagnosis. The K-adaptive partitioning algorithm was adopted to define subgroups with homogeneous survival. The combined R-ISS with PET/CT classified NDMM patients into four groups: R-ISS/PET stage I (*n* = 31; R-ISS I with FL ≤ 3), stage II (*n* = 156; R-ISS I with FL > 3 and R-ISS II with FL ≤ 3), stage III (*n* = 162; R-ISS II with FL > 3 and R-ISS III with FL ≤ 3), and stage IV (*n* = 31; R-ISS III with FL > 3). The 2-year overall survival rates for stages I, II, III, and IV were 96.7%, 89.8%, 74.7%, and 50.3%. The 2-year progression-free survival rates were 84.1%, 64.7%, 40.8%, and 17.1%, respectively. The new R-ISS/PET was successfully validated in an external cohort. This new system had a remarkable prognostic power for estimating the survival outcomes of patients with NDMM. This system helps discriminate patients with a good prognosis from those with a poor prognosis more precisely.

## Introduction

Multiple myeloma (MM) is a hematologic malignancy in which a single clone of plasma cell infiltrates the bone marrow (BM) and end-organs, thereby provoking morbidity and mortality [1]. A better understanding of MM biology and pathogenesis, in the aspects of BM tumor microenvironment and genetic alteration, has enabled the development of novel agents such as proteasome inhibitors (PI) and immunomodulatory drugs (IMiD) [2]. These agents have provided patients with increased response and prolonged survival rates [3, 4]. However, despite the therapeutic efficacy of the novel agents, the natural course of MM remains highly variable. Some patients experience refractory or rapid progression after adequate management, whereas others may live for more than ten years without disease progression. Establishing parameters for predicting survival heterogeneity is necessary for guiding treatment decision-making.

The International Myeloma Working Group (IMWG) suggests the use of the Revised International Staging System (R-ISS) in patients with newly diagnosed MM (NDMM) [5], in combination with the following parameters: (i) beta-2 microglobulin, (ii) serum albumin, (iii) serum lactate dehydrogenase (LDH), and (iv) cytogenetic abnormalities (CA) [6–8]. Owing to its simplicity and excellent predictive power, regardless of age and type of treatment, it has been widely used in real-world practices. However, more than half of the patients tend to be allocated to R-ISS stage II [9–11]. Furthermore, their prognosis is heterogeneous and can be separated by other discriminating variables [12]. In addition, patients with multiple bone lesions and extramedullary disease (EMD), which are regarded as high-risk features in MM, are not incorporated into the R-ISS [13–15].

Recently, the IMWG updated the diagnostic criteria of MM and recommends the use of new imaging techniques, including computed tomography (CT), magnetic resonance imaging (MRI), and ^18^F-fluorodeoxyglucose positron emission tomography/CT (^18^F-FDG PET/CT) to define the disease upon initial diagnosis [16]. Among these, ^18^F-FDG PET/CT is considered suitable for the assessment of metabolically active EMD in soft tissues along with FL in the bone and BM [17]. Many studies have demonstrated that abnormal ^18^F-FDG PET/CT results are strongly associated with negative patient outcomes [18]. Moreover, parameters such as the number of FL, maximum standardized uptake value (SUV_max_), and high metabolic tumor volume were suggested as surrogate markers for predicting overall survival (OS) and progression-free survival (PFS) [19–22].

Based on the clinical significance of imaging studies, it may be assumed that incorporating imaging results into the risk stratification system would enhance the degree of discriminating survival differences in MM. To the best of our knowledge, there have been only few attempts to combine high-risk features detected using novel imaging techniques with the risk stratification system. Thus, the present study aimed to design a new risk stratification system that includes information from ^18^F-FDG PET/CT results into the R-ISS in patients with NDMM. We report that the new risk stratification system could effectively stratify patients according to their survival outcomes.

## Methods

### Patients and treatment

The present study included NDMM patients who underwent ^18^F-FDG PET/CT upon diagnosis at 10 hospitals of the Korean Multiple Myeloma Working Party from September 2009 to March 2020. Other inclusion criteria include symptomatic MM and frontline treatment with PI and/or IMiD. The exclusion criteria were monoclonal gammopathy of undetermined significance, smoldering MM, and solitary plasmacytoma. Patients with hypermetabolic lesions caused by concomitant infection upon ^18^F-FDG PET/CT were also excluded.

Patients with good performance status (PS) and less than 65 years old, who achieved partial or better response after frontline therapy received upfront autologous stem cell transplantation following high-dose chemotherapy with melphalan with or without other cytotoxic agents. This study was approved by the Institutional Review Board of the Kyungpook National University Hospital (IRB No. 2020-03-070) and by each participating center according to the Declaration of Helsinki. All patients were exempted from informed consent for the scientific use of their data based on the Code of Federal Regulation.

### Initial diagnostic evaluation and response evaluation

The initial diagnostic evaluation included physical examination, baseline blood and urine tests, BM examination, and imaging studies. PS was determined by the Eastern Cooperative Oncology Group (ECOG) [23]. The laboratory tests included complete blood count, renal and liver function tests, serum calcium, LDH, beta-2 microglobulin, immunoglobulin, serum free light chain, serum protein electrophoresis with immunofixation, and urine analysis with electrophoresis and immunofixation. BM aspiration and biopsy were performed on the posterior iliac crest to measure the percentage of malignant plasma cells and to detect the subtype of MM. From the obtained BM specimen, CD138-positive cells were purified, and high-risk CA—deletion of 17p13, t(4;14)(p16;q32) and t(14;16)(q32;q23)—was tested using the interphase fluorescence in situ hybridization (iFISH) method [8]. Imaging studies included radiography, CT, ^18^F-FDG PET/CT, and/or MRI.

R-ISS stage I was defined as ISS stage I (serum beta2-microglobulin < 3.5 mg/L and serum albumin ≥ 3.5 g/dL) with standard-risk CA by iFISH and normal LDH level. R-ISS stage III was defined as ISS stage III (serum beta2-microglobulin ≥ 5.5 mg/L) with either high-risk CA or high LDH level. R-ISS stage II included all patients who did not have R-ISS stage I or III [5]. Response evaluation was performed according to the IMWG consensus criteria [24].

### ^18^F-FDG PET/CT evaluation

^18^F-FDG PET/CT imaging was performed using a Discovery ST FDG PET/CT system (GE Healthcare) on most hospitals. The patients fasted for at least 6 h before the intravenous administration of ^18^F-FDG [4.1–7.4 megabecquerel (MBq) per kg of body weight] to ensure a serum glucose level of < 7.2 mmol/L. At 60 ± 10 minutes after FDG administration, a low-dose CT scan was obtained without contrast enhancement for attenuation correction from the base of the skull to the proximal thighs. PET scans were acquired for the same anatomic sites. The images were reconstructed using a conventional iterative algorithm. Workstations (AW Volume Share) providing multiplanar reformatted images were used for image display and analysis.

FL is the focally discrete accumulation of malignant plasma cells in the bone or BM. ^18^F-FDG PET/CT showed that FL have a higher FDG uptake than the liver or the physiological BM. FL was described by number, location, and associated SUV_max_ values with or without any underlying identified bone lesions. In contrast, EMD is characterized by an FDG-avid lump harboring malignant plasma cells in soft tissues not contiguous to the bones. Moreover, it may have resulted from the hematogenous spread of the plasma cells.

### Patients in the external validation cohort

The independent data of NDMM patients who underwent ^18^F-FDG PET/CT were collected in one hospital from June 2006 to February 2021 as the validation cohort. Patients’ inclusion and exclusion criteria in the external validation cohort were the same as those in the original cohort.

### Statistical analysis

Data of patient characteristics are presented as proportions and medians. Continuous variables were compared using two-sample *t*-test or analysis of variance, while categorical data were compared using the chi-square test. Logistic regression test was used to identify the factors that affected treatment responses. PFS was defined as the time from diagnosis until disease progression or all-cause death. OS was defined as the time from diagnosis until the last follow-up or all-cause death. The Kaplan-Meier method was used to plot curves for PFS and OS, with group comparisons performed using a log-rank test. For measuring the goodness of risk, Harrell’s C-index was used. A C-index value near 0.7 indicates that the risk model is good at predicting survival outcomes. Prognostic factors affecting OS and PFS were evaluated using a Cox regression model. Factors with a *p*-value of less than 0.1 in the univariate analysis were entered in the multivariate analysis, and *p*-values of less than 0.05 were considered to indicate statistical significance. A K-partitioning algorithm was used to define new risk groups that would show significant differences in survival; this was carried out using the “kcaps” package in R. For statistical analyses, R statistical software 3.6.3 (the R foundation for Statistical Computing, Vienna, Austria; available at http://www.r-project.org) and SPSS version 20.0 (SPSS Inc. IBM Corp., Chicago) were used.

## Results

### Patient characteristics and treatment outcomes

A total of 405 patients with NDMM were initially assessed for eligibility. However, 25 patients were excluded for missing values or lost to follow-up (Fig. S1). Therefore, the data of 380 patients were included in the study. Of the patients, 207 (54.5%) were aged 65 years or older, and 198 (52.1%) were male. ECOG PS was 0 or 1 in 294 patients (77.4%) and LDH was increased in 106 patients (27.9%). Immunoglobulin isotypes, namely, IgG, A, M, D, and light chain type, were found in 216 (56.8%), 83 (21.8%), 3 (0.8%), 4 (1.1%), and 74 (19.5%) patients, respectively. CA by iFISH showed that 71 patients (18.7%) had high-risk CA. All patients received at least one novel agent as the frontline treatment. Furthermore, 307 (80.8%) patients received PI (bortezomib, carfilzomib, and ixazomib), and 188 (49.5%) patients received IMiD (thalidomide and lenalidomide) Both PI and IMiD were administered in 115 (30.3%) patients (Table S1). Regarding treatment response, complete response, very good partial response, and partial response (PR) were found in 103 patients (27.1%), 107 (28.2%), and 113 (29.7%), respectively. Moreover, 37 (9.7%) and 20 (5.3%) patients had stable disease and had progressive disease, respectively. The detailed patient characteristics and treatment outcomes are summarized in Table 1.

**Table 1:** Patient characteristics (*n* = 380).

Characteristics	No (%)
Age, median (range) years	66 (34–86)
≥ 65 years	207 (54.5)
Sex	
Male	198 (52.1)
Female	182 (47.9)
ECOG PS	
0–1	294 (77.4)
2–3	76 (20.0)
Unknown	10 (2.6)
Subtype	
Ig G	216 (56.8)
Ig A	83 (21.8)
Ig M	3 (0.8)
Ig D	4 (1.1)
Light chain type	74 (19.5)
Light chain	
Kappa	199 (52.4)
Lambda	177 (46.6)
LDH, increased	106 (27.9)
Albumin ≥ 3.5 g/dL	234 (61.6)
Beta-2-microglobulin ≥ 5.5 mg/L	140 (36.8)
CA by iFISH	
Standard risk	309 (81.3)
High-risk^a^	71 (18.7)
R-ISS	
I	78 (20.5)
II	230 (60.5)
III	72 (18.9)
EMD	51 (13.4)
FL on PET/CT	
≤ 3	181 (47.6)
> 3	199 (52.4)
Frontline therapy	
Proteasome inhibitors	307 (80.8)^b^
Immunomodulatory agents	188 (49.5)^c^
Autologous SCT	131 (34.5)
Response to frontline therapy	
Complete response	103 (27.1)
Very good partial response	107 (28.2)
Partial response	113 (29.7)
Stable disease	37 (9.7)
Progressive disease	20 (5.3)
Progression	180 (47.7)
Death	84 (22.1)

*ECOG PS* Eastern Cooperative Oncology Group performance status, *Ig* immunoglobulin, *LDH* lactate dehydrogenase, *CA* cytogenetic abnormalities, *iFISH* interphase fluorescent in situ hybridization, *R-ISS* Revised International Staging System, *EMD* extramedullary disease, *FL* focal lesions, *PET/CT* positron emission tomography/computed tomography, *SCT* stem cell transplantation.

^a^Presence of del (17p) and/or t (4;14) and/or t (14;16).

^b^Patients received at least one proteasome inhibitor among bortezomib, carfilzomib, or ixazomib. One hundred fifteen patients received combination therapy including immunomodulatory agent and were counted twice.

^c^Patients received at least one immunomodulatory agent among thalidomide or lenalidomide.

### Focal lesions on PET/CT and R-ISS

**Fig. 1:**
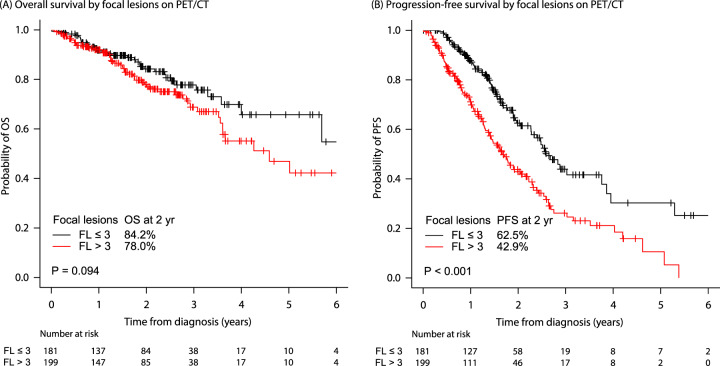
Survival rates according to the number of focal lesions on PET/CT. **A** Overall survival rates and **B** progression-free survival rates according to the focal lesions on PET/CT. Abbreviation: PET/CT positron emission tomography/computed tomography, FL focal lesions, OS overall survival, PFS progression-free survival.

**Fig. 2:**
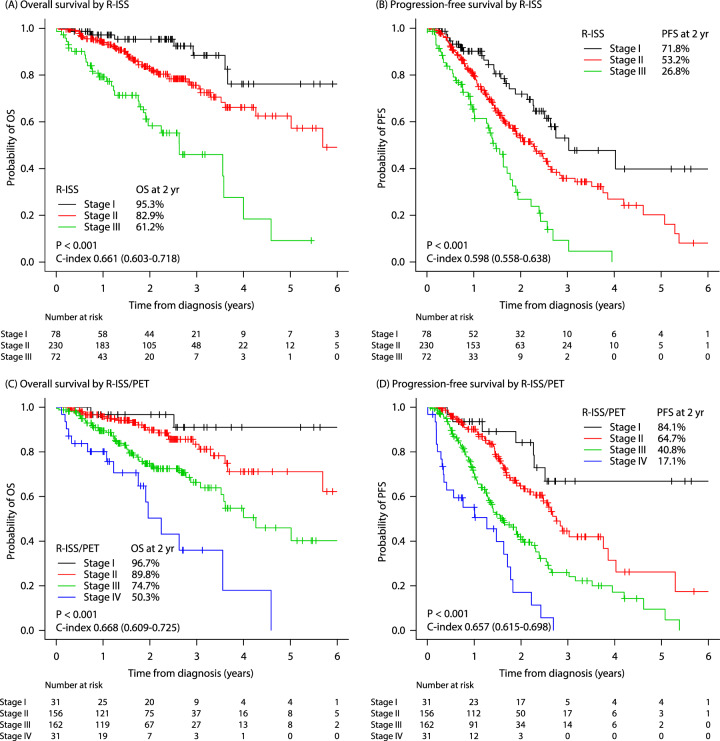
Comparison of the R-ISS and the R-ISS/PET survival curves. **A**, **B** Overall survival (OS) rates and progression-free survival (PFS) rates by the R-ISS. **C**, **D** OS and PFS rates by the R-ISS/PET. Abbreviation: R-ISS Revised International Staging System, R-ISS/PET Revised International Staging System/positron emission tomography.

During ^18^F-FDG PET/CT evaluation, more than three FL (FL > 3) were seen in 199 patients (52.4%) (Table 1). The median follow-up duration was 26 months (range 0.1–153 months). The 2-year OS rates were 84.2% (81.1–87.3) and 78.0% (74.6–81.4) for patients with FL ≤ 3 and FL > 3, respectively (*p* = 0.094), while the 2-year PFS rates were 62.5% (58.0–66.8) and 42.9% (38.8–47.0) for patients with FL ≤ 3 and FL > 3, respectively (*p* < 0.001) (Fig. 1). In the R-ISS group, 78 patients (20.5%) had R-ISS stage I, 230 (60.5%) were stage II, and 72 (18.9%) were stage III (Table 1). The 2-year OS rates were 95.3% (92.6–98.0), 82.9% (80.0–85.8), and 61.2% (54.3–68.1) for R-ISS stages I, II, and III, respectively (*p* < 0.001) (Fig. 2A). The PFS rates at 2 years were 71.8% (65.6–78.0), 53.2% (49.3–57.1), and 26.8% (19.9–33.7) for R-ISS stages I, II, and III, respectively (*p* < 0.001) (Fig. 2B).

In the multivariate analysis including the R-ISS and covariates: age, sex, ECOG PS, EMD, and FL > 3, the R-ISS was significantly associated with OS, in terms of stage II (hazard ratio [HR] 2.24, 95% confidence interval [CI] 0.95–5.32; *p* = 0.066) and stage III (HR 6.61, 95% CI 2.68–16.29; *p* < 0.001) versus stage I. Other factors affecting OS were ECOG PS (HR 1.90, 95% CI 1.18–3.05; *p* = 0.008) and EMD (HR 1.98, 95% CI 1.18–3.32; *p* = 0.010) (Table S2). Furthermore, the R-ISS was also significantly associated with PFS in stage II (HR 1.75, 95% CI 1.11–2.78; *p* = 0.017) and stage III (HR 4.23, 95% CI 2.50–7.17; *p* < 0.001) versus stage I. Other factors affecting PFS were FL > 3 by ^18^F-FDG PET/CT (HR 2.32, 95% CI 1.70–3.18; *p* < 0.001) and age ≥ 65 years (HR 1.43, 95% CI 1.05–1.96; *p* = 0.025) (Table S3).

### Combination of focal lesions on PET/CT and R-ISS

In the present study, the number of FL on ^18^F-FDG PET/CT was selected as a surrogate factor that can represent the prognostic implication of bone lesions in MM. Therefore, FL > 3 was combined with R-ISS to design the new risk stratification system R-ISS/PET. For its classification, the K-adaptive partitioning algorithm that can provide a statistically optimized combination of R-ISS and FL was performed. As a result, the following four groups were identified (Table 2): (i) 31 patients (8.2%) with R-ISS/PET stage I (R-ISS I with FL ≤ 3); (ii) 156 (41.1%) with stage II (R-ISS I with FL > 3 and R-ISS II with FL ≤ 3); (iii) 162 (42.6%) with stage III (R-ISS II with FL > 3 and R-ISS III with FL ≤ 3); and (iv) 31 (8.2%) with stage IV (R-ISS III with FL > 3).

**Table 2:** New risk stratification system including the number of focal lesions on PET/CT with the R-ISS.

R-ISS/PET	R-ISS	No of FL	No (%)	OS at 2 years % (95% CI)	PFS at 2 years % (95% CI)
Stage I	I	≤ 3	31 (8.2)	96.7 (93.4–100.0)	84.1 (76.6–91.6)
Stage II	I	> 3	156 (41.1)	89.8 (86.9–92.7)	64.7 (60.0–69.4)
	II	≤ 3			
Stage III	II	> 3	162 (42.6)	74.7 (70.8–78.6)	40.8 (36.2–45.4)
	III	≤ 3			
Stage IV	III	> 3	31 (8.2)	50.3 (38.4–62.2)	17.1 (8.5–25.7)

*PET/CT* positron emission tomography/computed tomography, *R-ISS* Revised International Staging System, *R-ISS/PET* Revised International Staging System/positron emission tomography, *FL* focal lesions, *OS* overall survival, *PFS* progression-free survival.

The new R-ISS/PET model successfully distinguished the patients into subgroups with regard to survival outcomes. The 2-year OS rates were 96.7% (93.4–100.0), 89.8% (89.6–92.7), 74.7% (70.8–78.6), and 50.3% (38.4–62.2) in R-ISS/PET stages I, II, III, and IV, respectively (*p* < 0.001) (Fig. 2C). The 2-year PFS rates were 84.1% (76.6–91.6), 64.7% (60.0–69.4), 40.8% (36.2–45.4), and 17.1 (8.5–25.7) in R-ISS/PET stages I, II, III, and IV, respectively (*p* < 0.001) (Fig. 2D). The C-index values were 0.668 (0.609–0.725) and 0.657 (0.615-0.698) for OS and for PFS, respectively (Fig. 2C, D). In the subgroup analyses, R-ISS/PET was identified as a prognostic factor for both OS and PFS, regardless of transplant eligibility (Fig. S2). Additionally, the prognostic role of the R-ISS/PET for survival outcomes was significantly confirmed in patients who received any type of treatment with either PI or IMiD (Fig. S3).

**Fig. 3:**
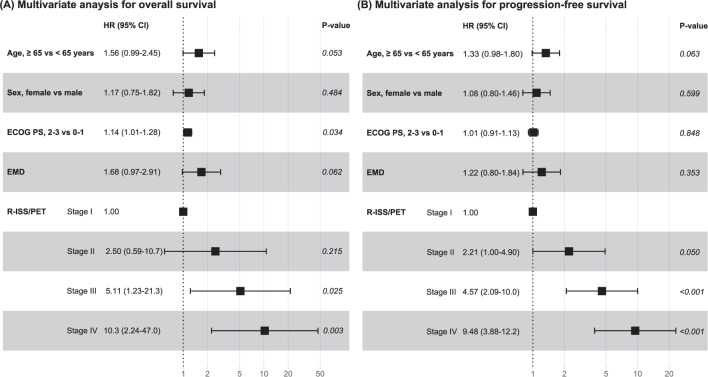
Multivariate analyses for overall survival and progression-free survival with the R-ISS/PET. Abbreviation: R-ISS/PET Revised International Staging System/positron emission tomography, HR hazard ratio, CI confidence interval, ECOG PS Eastern Cooperative Oncology Group performance status, EMD extramedullary disease.

In the univariate Cox analysis, we found that age ≥ 65, ECOG PS 2–3, EMD, and R-ISS/PET were associated with significantly poorer OS and PFS (Table S4). In the multivariate Cox analysis for OS, R-ISS/PET was a significant factor and could predict long-term outcomes with regard to OS (Fig. 3A): (i) stage II vs. I (HR 2.50, 95% CI 0.59–10.7; *p* = 0.215), (ii) stage III vs. I (HR 5.11, 95% CI 1.23–21.3; *p* = 0.025), and (iii) stage IV vs. I (HR 10.3, 95% CI 2.24–47.0; *p* = 0.003). It was also confirmed to predict the risk of progression (Fig. 3B): (i) stage II vs. I (HR 2.21, 95% CI 1.00–4.90; *p* = 0.050), (ii) stage III vs. I (HR 4.57, 95% CI 2.09–10.0; *p* < 0.001), and (iii) stage IV vs. I (HR 9.48, 95% CI 3.88–12.2; *p* < 0.001). The R-ISS/PET also showed distinct treatment response rates following frontline therapy according to stages. The response rates of achieving PR or better were 90.3% (*n* = 28 of 31), 91.7% (*n* = 143 of 156), 82.1% (*n* = 133 of 162), and 61.3% (*n* = 19 of 31) in R-ISS/PET stages I, II, III, and IV, respectively (Fig. S4). In the multivariate analysis, the new model was effective in predicting treatment response following frontline therapy (*p* = 0.001) (Table S5).

### External validation of the R-ISS/PET risk stratification system

**Fig. 4:**
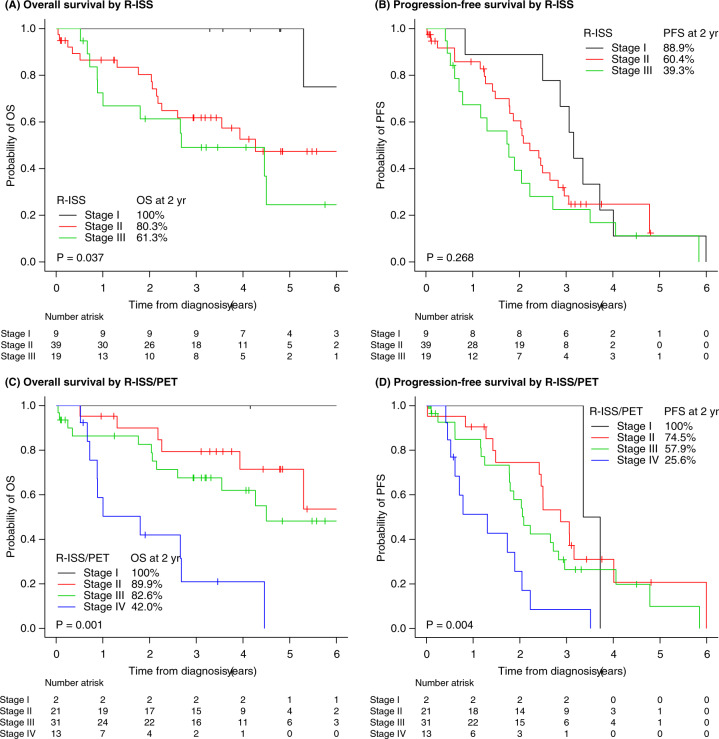
Comparison of the R-ISS and the R-ISS/PET survival curves in the external validation cohort. **A**, **B** Overall survival (OS) rates and progression-free survival (PFS) rates by the R-ISS. **C**, **D** OS and PFS rates by the R-ISS/PET in the external validation cohort. Abbreviation: *R-ISS* Revised International Staging System, *R-ISS/PET* Revised International Staging System/positron emission tomography.

We performed an external validation test to confirm the reproducibility of the new R-ISS/PET model. Sixty-seven patients in the external validation cohort had similar baseline characteristics as those of the 380 patients in the original cohort. The 2-year OS rates for R-ISS stages I, II, and III in the external validation cohort were 100%, 80.3% (73.6–87.0), and 61.3% (49.8–72.8), respectively (*p* = 0.037) (Fig. 4A). The 2-year PFS rates for R-ISS stages I, II, and III were 88.9% (78.4–99.4), 60.4% (51.8–69.0), and 39.3% (27.8–50.8), respectively (*p* = 0.268) (Fig. 4B). In the R-ISS/PET group, 2 (2.6%), 21 (27.6%), 31 (40.8%), and 13 (17.1%) patients were classified as R-ISS/PET stages I, II, III, and IV, respectively (Table S6). The 2-year OS rates for each of the R-ISS/PET stage I, II, III, and IV, were 100%, 89.9% (83.1–96.7), 82.6% (75.5–89.7), and 42.0% (27.7–56.3), respectively (*p* = 0.001) (Fig. 4C). The PFS rates at 2 years for R-ISS/PET stages I, II, III, and IV were 100%, 74.5% (64.6–84.4), 57.9% (48.2–67.6), and 25.6% (12.9–38.3), respectively (*p* = 0.004) (Fig. 4D).

## Discussion

In this study, FL > 3 on ^18^F-FDG PET/CT was a reliable imaging parameter for predicting poor survival outcomes and was therefore incorporated into the R-ISS. The new risk stratification model R-ISS/PET can categorize NDMM patients into four risk groups and can clearly stratify these patients according to survival differences. It was successfully validated in the external cohort. Notably, the new R-ISS/PET could discriminate patients with excellent prognosis from those with dismal prognosis more precisely. The new model was also applicable to each subgroup of patients with regard to transplant eligibility and frontline treatment.

R-ISS successfully divided patients with NDMM into risk groups. However, there are limitations in that it could not reflect the status of skeletal involvement, which was associated with an increased morbidity and mortality of the patients. Several studies that use new imaging modalities have demonstrated the prognostic role of FL in MM. More than one FL on MRI in patients with asymptomatic MM were proved significant for an increased risk of progression to symptomatic disease [13, 25]. Moreover, the viable FL and EMD can be easily assessed by ^18^F-FDG PET/CT [17]. More than three FL detected by ^18^F-FDG PET/CT upon initial diagnosis were consistently documented to be associated with inferior survival outcomes [19, 20].

In the present study, ^18^F-FDG PET/CT was performed to evaluate the intra- and extramedullary involvement of MM upon diagnosis. We identified that FL > 3 on ^18^F-FDG PET/CT was strongly associated with adverse prognosis. The mechanisms underlying FL contribution to disease progression and dismal survival of patients have not been comprehensively elucidated. A few studies reported that the genomic features of a certain FL were found to be different from those of other FL in the bone or BM [26, 27]. This implies that the sub-clones associated with disease progression or drug resistance may co-exist upon diagnosis, and are not emergent from mutations acquired during treatments [28]. Thus, it could be cautiously assumed that the high number of FL might represent the sweeping existence of sub-clones harboring adverse genes, which suggests that the number of FL can be applied as a surrogate marker for predicting prognosis.

To develop a new risk stratification system, we combined FL on ^18^F-FDG PET/CT with each R-ISS stage. Therefore, six subgroups were identified. These subgroups were categorized into four stages using a K-partitioning algorithm in accordance with the risk score boundaries that would show significant differences in survival outcomes. The application of the new risk model to the patients revealed that R-ISS/PET could predict the long-term prognosis of patients regarding OS and PFS. In particular, this model could aid in categorizing patients with R-ISS stage II, which accounted for more than half of the NDMM patients and was considered clinically heterogeneous according to the high-risk features detected on ^18^F-FDG PET/CT [9–12]. This model could show prominent differences in survival outcomes between patients with an excellent prognosis and those with dismal prognosis. R-ISS/PET was also found to be effective in estimating the response rate of frontline therapy.

There are some limitations to the present study. We retrospectively reviewed the medical records of patients from ten hospitals. Some centers used different PET/CT machines. Therefore, inter-hospital standardization of the imaging interpretation of ^18^F-FDG PET/CT could not be performed in all centers. False-positive results of ^18^F-FDG PET/CT must altogether be considered under certain conditions such as infection or diabetes. Regardless, the procedure for performing ^18^F-FDG PET/CT followed the currently recommended preparation protocol. Aside from these technical issues, some patients with extensive bone involvement could have a negative result on ^18^F-FDG PET/CT because myeloma cells might have low expression of hexokinase-2, which is involved in the glycolysis of FDG in malignant cells [29]. Although new high-risk FISH markers, such as t(14;20), gain(1q21), and del(1p32) have been identified, they have not been used in our model [8, 30]. Therefore, a prospective study that can overcome the mentioned limitations is required to validate the new risk stratification system.

In conclusion, recent advances in imaging techniques have provided in-depth information on patients with NDMM with regard to the risk of survival. The new R-ISS/PET combination system enabled a more precise prediction of different survival groups among patients with NDMM. Thus, this model is applicable for identifying heterogeneous manifestations of clinical MM.

## Supplementary information

Supplementary material
