# Supplementary material for: Development of a new risk stratification system for patients with newly diagnosed multiple myeloma using R-ISS and 18F-FDG PET/CT
Source: Blood Cancer J. 2021 Dec 1;11(12):190. doi: 10.1038/s41408-021-00577-2 (PMC8636569; doi:10.1038/s41408-021-00577-2)
Supplement: Supplementary file 1 — Supplementary material [file 41408_2021_577_MOESM1_ESM.docx]

**Table S1. Frontline treatment**

| Transplant-eligible (n = 173) | No (%) |
| --- | --- |
| VTD | 105 (60.7) |
| CTD | 48 (27.7) |
| VCD | 20 (11.6) |
| Transplant-ineligible (n = 207) |  |
| VMP | 165 (79.7) |
| RD | 25 (12.1) |
| VTD | 6 (2.9) |
| IRD | 4 (1.9) |
| KMP | 3 (1.4) |
| D-VMP | 3 (1.4) |
| VCD | 1 (0.5) |

Abbreviations: VTD, bortezomib / thalidomide / dexamethasone; CTD, cyclophosphamide / thalidomide / dexamethasone; VCD, bortezomib / cyclophosphamide / dexamethasone; VMP, bortezomib / melphalan / prednisolone; RD, lenalidomide / dexamethasone; IRD, ixazomib / lenalidomide / dexamethasone; KMP, carfilzomib / melphalan / prednisolone; D-VMP, daratumumab / bortezomib / melphalan / prednisolone

**Table S2. Factors affecting overall survival rate with the R-ISS**

|  | **Univariate** | |  | **Multivariate** | |
| --- | --- | --- | --- | --- | --- |
|  | **HR (95% CI)** | ***p*-value** |  | **HR (95% CI)** | ***p*-value** |
| Age, ≥ 65 vs < 65 years | 1.71 (1.10-2.67) | 0.018 |  |  |  |
| Sex, female vs male | 1.00 (0.65-1.53) | 0.985 |  |  |  |
| ECOG PS, 2-3 vs 0-1 | 2.29 (1.44-3.66) | 0.001 |  | 1.90 (1.18-3.05) | 0.008 |
| High-risk CA | 1.49 (0.86-2.58) | 0.157 |  |  |  |
| EMD | 2.49 (1.50-4.12) | <0.001 |  | 1.98 (1.18-3.32) | 0.010 |
| FL on PET/CT, > 3 vs ≤ 3 | 1.45 (0.94-2.25) | 0.096 |  |  |  |
| R-ISS |  | <0.001 |  |  | <0.001 |
| I | 1.00 |  |  | 1.00 |  |
| II | 2.49 (1.12-5.52) | 0.025 |  | 2.24 (0.95-5.32) | 0.066 |
| III | 7.69 (3.36-17.63) | <0.001 |  | 6.61 (2.68-16.29) | <0.001 |
| Frontline therapy |  |  |  |  |  |
| Non-PI | 1.00 |  |  |  |  |
| PI | 1.19 (0.71-2.01) | 0.517 |  |  |  |

Abbreviations: R-ISS, Revised International Staging System; HR, hazard ratio; CI, confidence interval; ECOG PS, Eastern Cooperative Oncology Group performance status; CA, cytogenetic abnormalities; EMD, extramedullary disease**;** FL, focal lesions; PET/CT, positron emission tomography/computed tomography; PI, proteasome inhibitors

**Table S3. Factors affecting progression-free survival rate with the R-ISS**

|  | **Univariate** | |  | **Multivariate** | |
| --- | --- | --- | --- | --- | --- |
|  | **HR (95% CI)** | ***p*-value** |  | **HR (95% CI)** | ***p*-value** |
| Age, ≥ 65 vs < 65 years | 1.43 (1.06-1.92) | 0.018 |  | 1.43 (1.05-1.96) | 0.025 |
| Sex, female vs male | 0.96 (0.72-1.29) | 0.799 |  |  |  |
| ECOG PS, 2-3 vs 0-1 | 1.52 (1.06-2.17) | 0.022 |  |  |  |
| High-risk CA | 1.19 (0.81-1.76) | 0.376 |  |  |  |
| EMD | 1.77 (1.20-2.63) | 0.004 |  |  |  |
| FL on PET/CT, > 3 vs ≤ 3 | 1.99 (1.47-2.69) | <0.001 |  | 2.32 (1.70-3.18) | <0.001 |
| R-ISS |  | <0.001 |  |  | <0.001 |
| I | 1.00 |  |  | 1.00 |  |
| II | 1.87 (1.20-2.90) | 0.006 |  | 1.75 (1.11-2.78) | 0.017 |
| III | 3.84 (2.32-6.35) | <0.001 |  | 4.23 (2.50-7.17) | <0.001 |
| Frontline therapy |  |  |  |  |  |
| Non-PI | 1.00 |  |  |  |  |
| PI | 1.10 (0.78-1.55) | 0.619 |  |  |  |

Abbreviations: R-ISS, Revised International Staging System; HR, hazard ratio; CI, confidence interval; ECOG PS, Eastern Cooperative Oncology Group performance status; CA, cytogenetic abnormalities; EMD, extramedullary disease; FL, focal lesions; PET/CT, positron emission tomography/computed tomography; PI, proteasome inhibitors

**Table S4. Univariate analysis for overall survival and progression-free survival with the R-ISS/PET**

|  | **Overall survival** | |  | **Progression-free survival** | |
| --- | --- | --- | --- | --- | --- |
|  | **HR (95% CI)** | ***p*-value** |  | **HR (95% CI)** | ***p*-value** |
| Age, ≥ 65 vs < 65 years | 1.71 (1.10-2.67) | 0.018 |  | 1.43 (1.06-1.92) | 0.018 |
| Sex, female vs male | 1.00 (0.65-1.53) | 0.985 |  | 0.96 (0.72-1.29) | 0.799 |
| ECOG PS, 2-3 vs 0-1 | 2.29 (1.44-3.66) | 0.001 |  | 1.52 (1.06-2.17) | 0.022 |
| High-risk CA | 1.49 (0.86-2.58) | 0.157 |  | 1.19 (0.81-1.76) | 0.376 |
| EMD | 2.49 (1.50-4.12) | <0.001 |  | 1.77 (1.20-2.63) | 0.004 |
| R-ISS/PET |  | <0.001 |  |  | <0.001 |
| I | 1.00 |  |  | 1.00 |  |
| II | 2.48 (0.58-10.6) | 0.216 |  | 2.27 (1.03-5.02) | 0.042 |
| III | 5.80 (1.41-23.9) | 0.015 |  | 4.77 (2.19-10.4) | <0.001 |
| IV | 14.6 (3.31-63.9) | <0.001 |  | 10.6 (4.50-25.1) | <0.001 |
| Frontline therapy |  |  |  |  |  |
| Non-PI | 1.00 |  |  | 1.00 |  |
| PI | 1.19 (0.71-2.01) | 0.517 |  | 1.10 (0.78-1.55) | 0.619 |

Abbreviations: R-ISS/PET, Revised International Staging System/positron emission tomography; HR, hazard ratio; CI, confidence interval; ECOG PS, Eastern Cooperative Oncology Group performance status; CA, cytogenetic abnormalities; EMD, extramedullary disease; PI, proteasome inhibitors

**Table S5. Factors affecting treatment response after the frontline therapy**

|  | **Univariate** | |  | **Multivariate** | |
| --- | --- | --- | --- | --- | --- |
|  | **OR (95% CI)** | ***p*-value** |  | **OR (95% CI)** | ***p*-value** |
| Age, ≥ 65 vs < 65 years | 0.41 (0.22-0.77) | 0.005 |  | 0.45 (0.24-0.84) | 0.012 |
| Sex, female vs male | 0.87 (0.50-1.53) | 0.625 |  |  |  |
| ECOG PS, 2-3 vs 0-1 | 0.42 (0.22-0.78) | 0.006 |  |  |  |
| LDH, increased | 0.47 (0.26-0.84) | 0.011 |  |  |  |
| High-risk CA | 1.48 (0.67-3.29) | 0.331 |  |  |  |
| EMD | 0.45 (0.22-0.91) | 0.027 |  |  |  |
| FL on PET/CT, > 3 vs ≤ 3 | 0.41 (0.22-0.76) | 0.004 |  |  |  |
| R-ISS |  | 0.045 |  |  |  |
| I | 1.00 |  |  |  |  |
| II | 0.59 (0.25-1.39) | 0.227 |  |  |  |
| III | 0.32 (0.12-0.82) | 0.018 |  |  |  |
| R-ISS/PET |  | <0.001 |  |  | 0.001 |
| I | 1.0 |  |  | 1.0 |  |
| II | 1.18 (0.31-4.41) | 0.807 |  | 1.23 (0.33-4.66) | 0.757 |
| III | 0.49 (0.14-1.73) | 0.268 |  | 0.54 (0.15-1.91) | 0.336 |
| IV | 0.17 (0.04-0.68) | 0.013 |  | 0.19 (0.05-0.77) | 0.020 |
| Frontline therapy |  |  |  |  |  |
| Non-PI | 1.00 |  |  |  |  |
| PI | 1.01 (0.49-2.06) | 0.985 |  |  |  |

Abbreviations: OR, odds ratio; CI, confidence interval; ECOG PS, Eastern Cooperative Oncology Group performance status; LDH, lactate dehydrogenase; CA, cytogenetic abnormalities; EMD, extramedullary disease; FL, focal lesions; PET/CT, positron emission tomography/computed tomography; R-ISS, Revised International Staging System; R-ISS/PET, Revised International Staging System/ positron emission tomography; PI, proteasome inhibitors

**Table S6. Patient characteristics in the external validation cohort (n= 67)**

| **Characteristics** | **No (%)** |
| --- | --- |
| Age, median (range) years | 65 (32 – 83) |
| ≥ 65 years | 37 (55.2) |
| Sex |  |
| Male | 39 (58.2) |
| Female | 28 (41.8) |
| ECOG PS |  |
| 0-1 | 37 (55.2) |
| 2-3 | 29 (43.3) |
| Unknown | 1 (1.5) |
| LDH, increased | 24 (35.8) |
| Albumin ≥ 3.5g/dL | 36 (53.7) |
| Beta2-microglobulin ≥ 5.5mg/L | 27 (40.3) |
| CA by iFISH |  |
| Standard risk | 46 (68.7) |
| High-risk^*^ | 21 (31.3) |
| R-ISS |  |
| I | 9 (13.4) |
| II | 39 (58.2) |
| III | 19 (28.4) |
| EMD | 8 (11.9) |
| FL on PET/CT |  |
| ≤ 3 | 22 (32.8) |
| > 3 | 45 (67.2) |
| R-ISS/PET |  |
| I | 2 (2.6) |
| II | 21 (27.6) |
| III | 31 (40.8) |
| IV | 13 (17.1) |
| Frontline therapy |  |
| Proteasome inhibitors | 50 (74.6) ^†^ |
| Immunomodulatory agents | 30 (44.8) ^‡^ |
| Autologous SCT | 32 (47.8) |
| Response to frontline therapy |  |
| Complete response | 13 (19.4) |
| Very good partial response | 9 (13.4) |
| Partial response | 29 (43.3) |
| Stable disease | 13 (19.4) |
| Progressive disease | 3 (4.5) |
| Progression | 51 (76.1) |
| Death | 28 (41.8) |

Abbreviations: ECOG PS, Eastern Cooperative Oncology Group performance status; Ig, immunoglobulin; LDH, lactate dehydrogenase; CA, cytogenetic abnormalities; iFISH, interphase fluorescent in situ hybridization; R-ISS, Revised-International Staging System; EMD, extramedullary disease; FL, focal lesions; PET/CT, positron emission tomography/computed tomography; R-ISS/PET, Revised International Staging System/ positron emission tomography; SCT, stem cell transplantation

^*^ Presence of del (17p) and/or t (4;14) and/or t (14;16)

^†^ Patients received at least one proteasome inhibitor among bortezomib, carfilzomib, or ixazomib. Sixteen patients received combination therapy including immunomodulatory agent and were counted twice.

^‡^ Patients received at least one immunomodulatory agent among thalidomide or lenalidomide. Sixteen patients received combination therapy including proteasome inhibitor and were counted twice.

**Figure S1. CONSORT flow diagram**


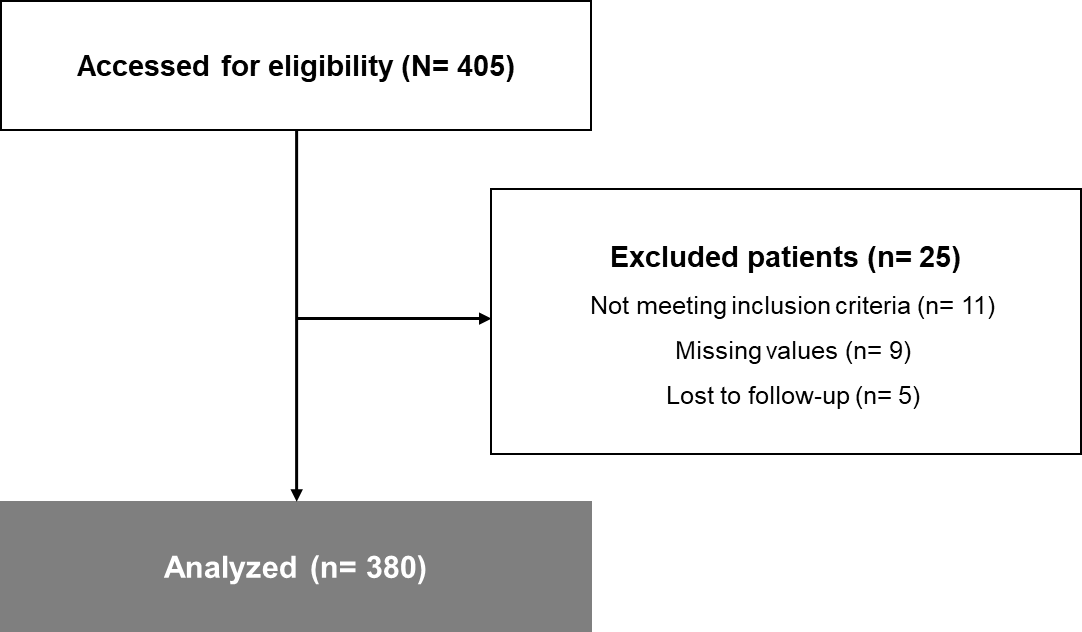


**Figure S2. Survival rates according to the R-ISS/PET by transplant eligibility**


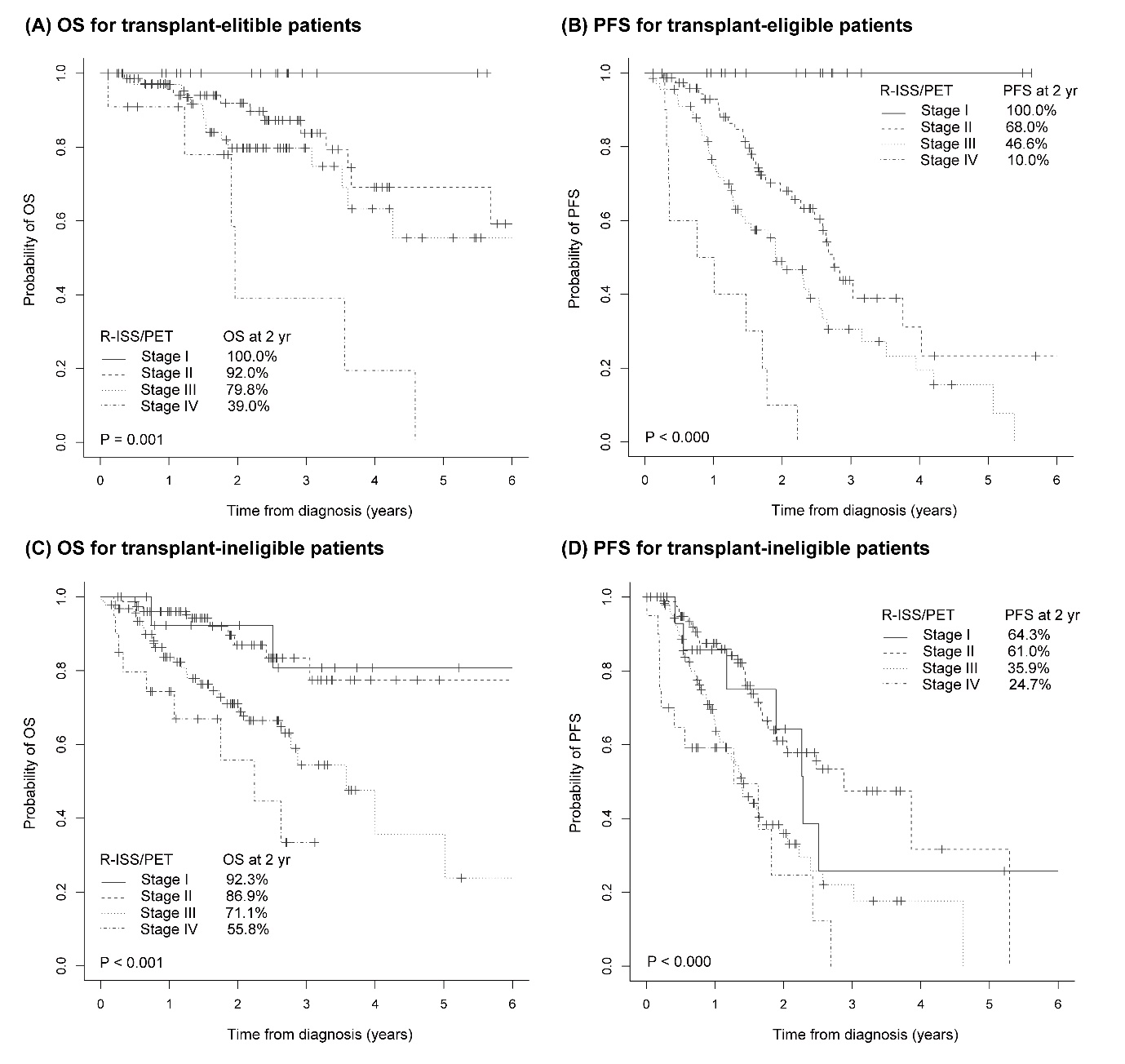


R-ISS/PET, Revised International Staging System/positron emission tomography; OS, overall survival; PFS, progression-free survival

**Figure S3. Survival rates according to the R-ISS/PET by frontline treatments**


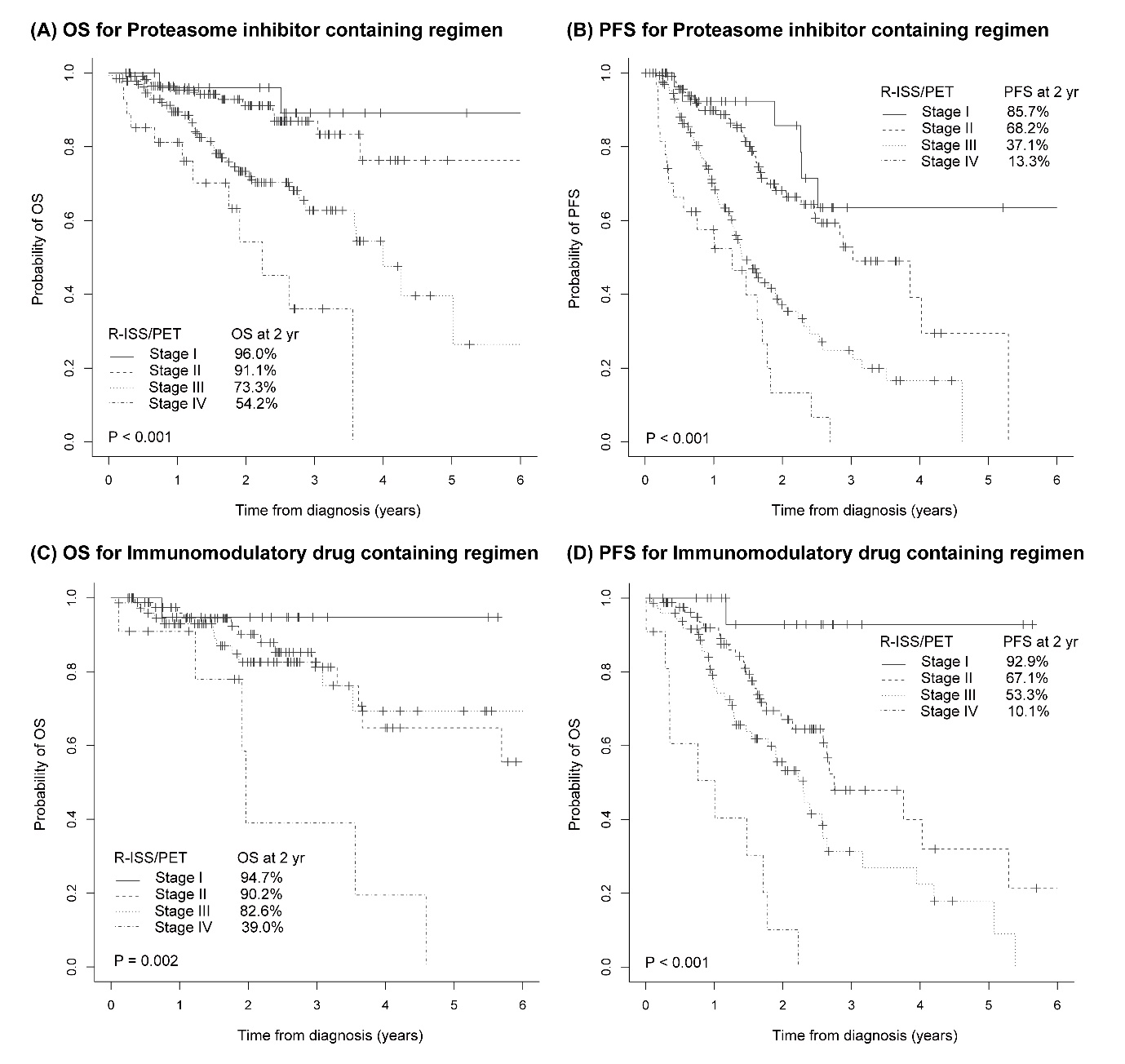


R-ISS/PET, Revised International Staging System/positron emission tomography; OS, overall survival; PFS, progression-free survival

**Figure S4. Treatment response after frontline treatment**


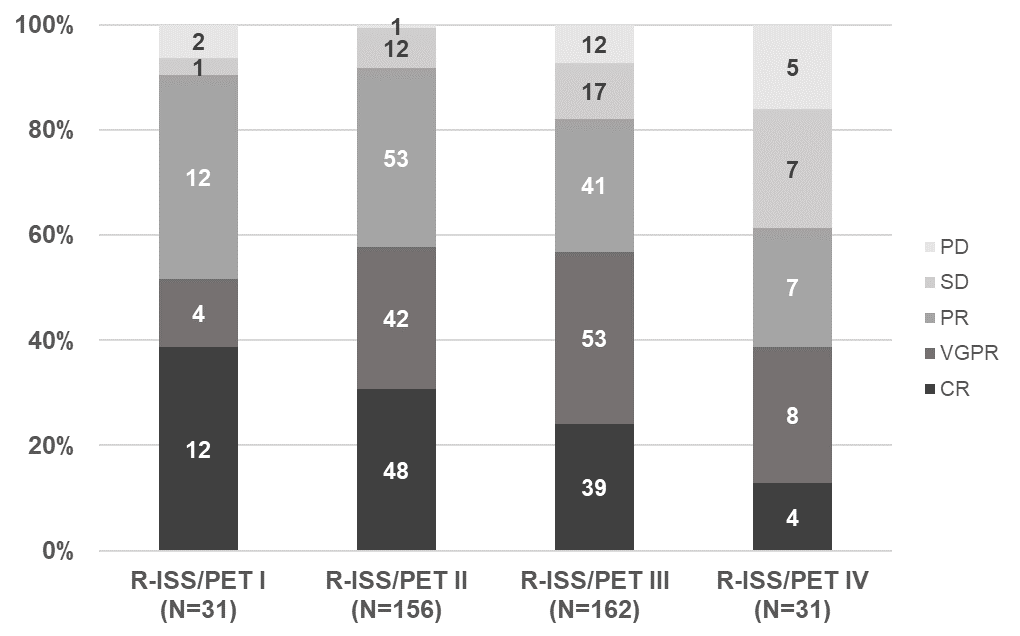


The response rate of achieving partial response or better were 90.3% (n=28 of 31), 91.7% (n=143 of 156), 82.1% (n=133 of 162), and 61.3% (n=19 of 31) in R-ISS/PET stage I, II, III, and IV, respectively.

Abbreviations: R-ISS/PET, Revised International Staging System/positron emission tomography; CR, complete response; VGPR, very good partial response; PR, partial response; MR, minimal response; SD, stable disease; PD, progressive disease
